# Clinical Profiles and Factors Associated with Death in Adults with Dengue Admitted to Intensive Care Units, Minas Gerais, Brazil

**DOI:** 10.1371/journal.pone.0129046

**Published:** 2015-06-19

**Authors:** Frederico Figueiredo Amâncio, Tiago Pires Heringer, Cristina da Cunha Hueb Barata de Oliveira, Liliane Boaventura Fassy, Frederico Bruzzi de Carvalho, Daniela Pagliari Oliveira, Claudio Dornas de Oliveira, Fernando Otoni Botoni, Fernanda do Carmo Magalhães, José Roberto Lambertucci, Mariângela Carneiro

**Affiliations:** 1 Pós-graduação em Ciências da Saúde: Infectologia e Medicina Tropical, Faculdade de Medicina, Universidade Federal de Minas Gerais, Belo Horizonte, Minas Gerais, Brazil; 2 Hospital João XXIII, Fundação Hospitalar do Estado de Minas Gerais, Belo Horizonte, Brazil; 3 Hospital Cesar Leite, Manhuaçu, Minas Gerais, Brazil; 4 Faculdade de Medicina, Universidade Federal do Triângulo Mineiro, Uberaba, Minas Gerais, Brazil; 5 Hospital Dr. Moises Magalhaes Freire, Pirapora, Minas Gerais, Brazil; 6 Hospital Eduardo de Menezes, Fundação Hospitalar do Estado de Minas Gerais, Belo Horizonte, Minas Gerais, Brazil; 7 Hospital Odilon Behrens, Belo Horizonte, Minas Gerais, Brazil; 8 Hospital Santa Casa de Belo Horizonte, Belo Horizonte, Minas Gerais, Brazil; 9 Faculdade de Medicina, Universidade Federal de Minas Gerais, Belo Horizonte, Brazil; 10 Hospital Risoleta Tolentino Neves, Belo Horizonte, Minas Gerais, Brazil; 11 Departamento de Parasitologia, Instituto de Ciências Biológicas, Universidade Federal de Minas Gerais, Belo Horizonte, Minas Gerais, Brazil; University of Rochester, UNITED STATES

## Abstract

The purpose of our study was to describe the clinical profile of dengue-infected patients admitted to Brazilian intensive care units (ICU) and evaluate factors associated with death. A longitudinal, multicenter case series study was conducted with laboratory-confirmed dengue patients admitted to nine Brazilian ICUs situated in Minas Gerais state, southeastern Brazil from January 1, 2008, to December 31, 2013. Demographic, clinical and laboratory data; disease severity scores; and mortality were evaluated. A total of 97 patients were studied. The in-ICU and in-hospital mortality rates were 18.6% and 19.6%, respectively. Patients classified as having severe dengue according to current World Health Organization classifications showed an increased risk of death in a univariate analysis. Nonsurvivors were older, exhibited lower serum albumin concentrations and higher total leukocyte counts and serum creatinine levels. Other risk factors (vomiting, lethargy/restlessness, dyspnea/respiratory distress) were also associated with death in a univariate analysis. Multivariate analysis indicated that in-hospital mortality was significantly associated with Acute Physiology and Chronic Health Evaluation II and the Sequential Organ Failure Assessment score. The ICU and in-hospital mortality observed in this study were higher than values reported in similar studies. An increased frequency of ICU admission due to severe organ dysfunction, higher severity indices and scarcity of ICU beds may partially explain the higher mortality.

## Introduction

Dengue is the most important arbovirosis in the world, with 2.5 billion people at risk and 50 million new cases every year. The World Health Organization (WHO) estimates that twenty thousand people die from dengue every year [1,2]. Most cases and deaths occur in developing countries and are managed in primary care settings or general clinical wards; however, a significant proportion of severe cases require intensive care [1,3]. Few studies have addressed the clinical and laboratory aspects of dengue cases treated in intensive care units [4–6].

Currently, Brazil is the leading country in terms of the number of dengue cases reported worldwide [1]. The country has experienced an expansion of basic health assistance over the last 20 years, but there are still tertiary care centers that lack a sufficient number of hospital beds [7]. In developing countries, such as Brazil, where ICU beds can be a limited health resource, understanding the causes of dengue admissions and deaths could improve the management of critical dengue patients.

The recent epidemiology of dengue disease in Brazil is characterized by an increase in the distribution and severity of dengue cases [8]. The recent reintroduction of serotype 4 occurred in 2010 associated with the previous presence of the three other serotypes intensified co-circulation of multiples serotypes [8]. The country has a dengue marked seasonality with most of cases occurring from December to May. The highest incidence of cases has occurred in those aged 20–59 years old with higher risk of death among elderly [8–10].

Minas Gerais state is the second most populous state in Brazil (Brazilian Institute of Geography and Statistics—Instituto Brasileiro de Geografia e Estatística-IBGE 2014) [11], and an increased number of deaths from dengue have been reported here over the last decade. Since 2011, with the introduction of dengue serotype 4 in Minas Gerais state, all four serotypes now circulate, and, in 2013, a major epidemic occurred with approximately half a million dengue cases reported [9].

The purpose of this study was to describe the clinical aspects of dengue patients admitted to intensive care units and to identify the factors associated with death.

## Methods

### Study site

The study was carried out in Minas Gerais state, which is located in southeast Brazil and is the second most populous Brazilian state (19,597,330 inhabitants) (Brazilian Institute of Geography and Statistics—Instituto Brasileiro de Geografia e Estatística-IBGE 2013) [11]. Dengue is endemic in most parts of Minas Gerais, and two major epidemics occurred between 2008 and 2013; approximately 269,000 and 500,000 dengue cases were reported statewide in 2010 and 2013, respectively [9,10].

### Study design and subjects

This study is a longitudinal, multicenter case series study that included only adult patients (≥ 15 years) with laboratory-confirmed cases of dengue admitted to nine intensive care units (ICUs) in Minas Gerais state, Brazil, from January 1, 2008, to December 31, 2013 (Hospital Eduardo de Menezes, Fundação Hospitalar do Estado de Minas Gerais, Belo Horizonte; Hospital João XXIII, Fundação Hospitalar do Estado de Minas Gerais, Belo Horizonte; Hospital Cesar Leite, Manhuaçu; Hospital das Clínicas da Universidade Federal do Triângulo Mineiro, Uberaba; Hospital Dr. Moises Magalhaes Freire, Pirapora; Hospital Municipal Odilon Behrens, Belo Horizonte; Hospital Santa Casa de Belo Horizonte, Belo Horizonte; Hospital Risoleta Tolentino Neves, Belo Horizonte; Hospital Nossa Senhora Aparecida, Belo Horizonte). Only the first ICU admission was considered.

Laboratory-confirmed dengue cases included those with at least one of the following positive laboratory results: dengue polymerase chain reaction (PCR), dengue immunoglobulin-M (IgM), nonstructural protein 1 (NS1) antigen test, or viral isolation. The State Laboratory of Public Health (Fundacão Ezequiel Dias, Minas Gerais, Brazil) performed IgM ELISA using the Panbio, Brisbane, Australia and IgM-capture immunoenzymatic technique (MAC-ELISA) developed by Kuno et al. [12] for the detection of IgM antibodies. For NS1 detection, the Platelia dengue NS1 Ag assay (Bio-Rad) was employed. DENV were isolated on C3/36 cells and the virus serotype was identified by an immunofluorescence assay using monoclonal antibodies as described by Gubler et al. [13]. PCR were performed using RT-PCR with primers and methodology described previously by Lanciotti et al. [14].

According to the Brazilian dengue guideline, dengue suspected cases are defined as a febrile illness presenting with at least 2 clinical findings, including headache, retro-orbital pain, myalgia, arthralgia, rash, prostration or hemorrhagic manifestation [15]. For the purpose of this study, we first selected all suspected dengue cases that were treated in ICUs statewide. Afterward, from nine ICUs selected by convenience we included just laboratory confirmed cases as explained above.

This study was approved by the Universidade Federal de Minas Gerais Institutional Ethical Review Board and by the Ethical Review Boards of the hospitals that had one; the need to obtain a signed informed consent form from the participants was waived.

### Data collection

Investigators (AFF, FLB, HTP), who are all physicians with dengue experience, reviewed the medical records of patients using standardized forms. To facilitate comparability, this study was undertaken using similar definitions to those used previously in other studies [5,6]. Based on the patient medical records, the cause of each patient’s admission was assigned by the investigator to one of the following groups: **respiratory failure**, patients who required oxygen supplementation and/or mechanical ventilation related to a pulmonary dysfunction (i.e. noncardiogenic pulmonary edema and/or pneumonia); **neurological failure**, patients with an altered consciousness and/or seizures; **shock or hypotension due systemic inflammatory response syndrome [16]**, patients with hypotension or shock who required volume or vasopressor agents; **severe thrombocytopenia with or without minor bleeding manifestations**; **severe gastrointestinal bleeding**, hypotension or shock due to melena or hematemesis; **renal failure,** defined as the need for dialysis; **cardiac failure**, myocarditis, heart failure or pulmonary embolism; and **miscellaneous**, patients with any other cause. If more than one cause was identified, the investigator reported the primary cause that instigated the request for ICU treatment. Data were collected regarding age, sex, Charlson index [17], laboratory exam results (including serum albumin and transaminase levels during the first 24 hours of ICU admission), use of invasive mechanical ventilation and vasopressor agents, and length of the hospital and ICU stays. The Acute Physiology and Chronic Health Evaluation (APACHE) II score [18] and the Sequential Organ Failure Assessment (SOFA) score [19] were determined for each patient on the first ICU day. Each patient was classified upon ICU admission according WHO 1997 [20] and WHO 2009 criteria [21]. Definitions of dengue hemorrhagic fever (DHF) and grading of DHF were based on WHO 1997 criteria [20]. DHF patients had to fulfill the following criteria: (1) signs and symptoms compatible with dengue according to the WHO; (2) plasma leakage according to clinical or radiological evidence of fluid accumulation or increased hematocrit by at least 20% or hypoalbuminemia (albumin level < 3.5 g/dL); (3) hemorrhagic manifestations at any site or positive tourniquet test; (4) platelet count ≤ 100,000 cell/mm^3^. According to the WHO 2009 criteria [21], patients were classified as having dengue with warning signs if there were reports of abdominal pain or tenderness, vomiting, clinical fluid accumulation, mucosal bleeding, lethargy or restlessness, hepatomegaly and a rise in hematocrit concurrent with a rapid drop in platelet count. Severe dengue criteria included the following symptoms: (1) severe plasma leakage leading to shock or fluid accumulation with respiratory distress, (2) severe bleeding, (3) severe organ involvement or (4) transaminase levels ≥ 1000 units/L. We recorded survival at the time of ICU discharge and at hospital discharge.

### Statistical Analysis

A database was generated using EpiData (version 3.2, EpiData Association, Odense, Denmark) and analyzed using Statistical Package for Social Sciences software (SPSS, version 12.0). Continuous data were compared using the Student’s *t* test or the Wilcoxon rank-sum test. Categorical variables were analyzed using the chi-square or Fisher exact tests. The strength of association was evaluated using an odds ratio (OR) and a 95% confidence interval (CI). Variables associated with death from dengue at a significance level of 0.20 in the univariate analysis were included in the multivariate analysis. A step-by-step backward selection procedure was used to select the variables and to produce the final multivariate logistic regression models. Only the variables that showed significant associations (p < 0.05) with death from dengue remained in the final model. Final model fit was assessed using the Hosmer-Lemeshow test [22]. Receiver operating characteristic (ROC) curves were constructed for the continuous laboratory variables and severity scores [23].

## Results

### Descriptive

During the period of study, 370 patients with cases of suspected dengue (≥ 15 years) were admitted to public intensive care units statewide (S1 Table). A total of 97 laboratory-confirmed dengue patients were included in our study. All patients had just one admission to the ICU. The mean age was 42.6±20.3 years, ranging from 15 to 91 years old, and 49.5% of the patients were men. Shock or hypotension due to a systemic inflammatory response, severe thrombocytopenia with or without minor bleeding and respiratory failure were the main causes of admission, comprising 22 (22.7%), 22 (22.7%) and 21 (21.6%) patients, respectively. Fever, myalgia and headache were the most frequent dengue symptoms. Among the warning signs, abdominal pain and vomiting were the most common. Hemorrhagic manifestations were reported in 71 (73.2%) patients. Spontaneous skin hemorrhagic manifestations were reported in 29 patients, petechiae in 23 patients (23.7%), ecchymosis in nine patients (9.3%) and suffusions in 6 patients (6.2%). Mucosal bleeding was reported in 29 patients, gum bleeding in 13 (13.4%), epistaxis in 12 (12.4%) and hematuria in 9 (9.3%) (Table 1). Some patients presented more than one hemorrhagic manifestation. Tourniquet tests were reported for eight patients, and six of these were positive. The serum albumin concentration was available for 69 patients. Median serum albumin was 2.9 g/dL, and 56 (81.2%) patients had serum albumin levels of less than 3.5 g/dL.

**Table 1:** Characteristics of 97 laboratory-confirmed dengue patients admitted to ICUs from 2008–2013 in Minas Gerais, Belo Horizonte, Brazil.

Characteristics	Patients, *n* = 97
Age (years), mean (SD)	42.6 (20.3)
Male sex, n (%)	48 (49.5)
Main reason for ICU admission, n (%)	
Shock or hypotension due to SRIS	22 (22.7)
Severe thrombocytopenia with or without minor bleeding	22 (22.7)
Respiratory failure	21 (21.6)
Gastrointestinal bleeding	9 (9.3)
Neurological failure	5 (5.2)
Renal failure	5 (5.2)
Cardiac failure	3 (3.1)
Other	10 (10.2)
Comorbid conditions, n (%)	
Hypertension	28 (28.9)
Diabetes mellitus	14 (14.4)
Renal chronic disease	4 (4.1)
Smoking	9 (9.3)
Charlson Index, n (%)	
0	67 (69.1)
1	15 (15.5)
2	9 (9.2)
3	4 (4.1)
4	2 (2.1)
**Signs and symptoms, n (%)**	
Fever	94 (96.9)
Myalgia	91 (93.8)
Headache	66 (68.0)
Retro-orbital pain	49 (50.5)
Arthralgia	29 (29.9)
Rash	18 (18.6)
Diarrhea	18 (18.6)
Cough	13 (13.4)
Plasma leakage signs	
Cavitary effusion	28 (28.9)
Edema	12 (12.4)
Hematocrit increase	12 (12.4)
Warning signs	
Abdominal pain and/or tenderness	58 (59.8)
Vomiting	43 (44.3)
Hepatomegaly	14 (14.4)
Hypotension and/or syncope	49 (50.5)
Dyspnea or respiratory distress	42 (43.3)
Lethargy or restlessness	20 (20.6)
Oliguria	12 (12.4)
Hemorrhagic manifestations (any)	71 (73.2)
Mucosal bleeding	29 (29.9)
Spontaneous bleeding of the skin	29 (29.9)
Gastrointestinal bleeding	22 (22.7)
APACHE II score, median (IR)	11 (6–16.5)
SOFA score, median (IR)	4 (2–8)
Duration of ICU stay (days), median (IR)	2.0 (1.0–5.0)
Duration of hospital stay (days), median (IR)	6.0 (4.0–12.5)
Time from symptoms onset to hospitalization (days), median (IR)	4.0 (3.0–6.0)
Dengue according to WHO 2009 criteria at ICU admission, n (%)	
Dengue with warning signs	29 (29.9)
Severe dengue	68 (70.1)
Dengue according to WHO 1997 criteria at ICU admission, n (%)	
Dengue	50 (51.6)
Dengue hemorrhagic fever grade I and II	27 (27.8)
Dengue hemorrhagic fever grade III and IV	20 (20.6)
Mortality, n (%)	
In-ICU mortality	18 (18.6)
In-hospital mortality	19 (19.6)

SD, standard deviation; SRIS, systemic inflammatory response syndrome; APACHE, Acute Physiology and Chronic Health Evaluation; SOFA, Sequential Organ Failure Assessment; IR, interquartile range; WHO, World Health Organization; ICU, intensive care unit.

Treatments, events and interventions are shown in Table 2. Antibiotics were employed in 45 (45.4%) patients: 21 due to pneumonia, 13 due to unspecific sepsis, 5 due to urinary infection, 2 due to pharyngitis and the rest due to endocarditis (1), phlebitis (1), diverticulitis (1) and diarrhea (1). Twenty-two (22.7%) patients were treated for septic shock, and 14 (14.4%) were given steroids as adjuvant septic shock therapy during their ICU stay. Thirty patients required inotropic or vasopressor agents (30.9%), and 29 required mechanical ventilation (29.9%) during their ICU stay. Fifteen patients (15.5%) were on mechanical ventilation before ICU admission, and twelve (12.4) were using inotropic or vasopressor agents. Two patients had cardiorespiratory arrest before ICU admission.

**Table 2:** Laboratory results, events and interventions in the 97 dengue patients admitted to intensive care units in Minas Gerais, Brazil.

Characteristics	Patients (*n* = 97)
Laboratory values	
Albumin (g/dL), mean ± SD	2.8 ± 0.6
Platelet count, mean ± SD	95,217 ± 72,478
Leukocyte count, mean ± SD	7,671 ± 6,498
Hematocrit, mean ± SD	34.7 ± 6.9
Hemoglobin (g/dL), mean ± SD	11.7 ± 2.4
Bast cell (%), mean ± SD	5.2 ± 7.8
Creatinine (g/dL), median (IR)	0.9 (0.6–1.3)
AST (U/L), median (IR)	69 (31–197)
ALT (U/L), median (IR)	52 (30–126)
Cardiorespiratory arrest before ICU, n (%)	2 (2.1)
Vasopressor or inotropic support before ICU, n (%)	12 (12.4)
Mechanical ventilation before ICU, n (%)	15 (15.5)
Mechanical ventilation during ICU stay, n (%)	29 (29.9)
Vasopressor or inotropic support during ICU stay, n (%)	30 (30.9)
Hemodialysis, n (%)	12 (12.4)
Red blood cell transfusion, n (%)	12 (12.4)
Platelet transfusion, n (%)	23 (23.7)
Fresh frozen plasma transfusion, n (%)	14 (14.4)
Septic shock during ICU stay, n (%)	22 (22.7)
Steroid use, n (%)	14 (14.4)
Antibiotic treatment, n (%)	45 (45.4)

SD, standard deviation; IR, interquartile range; ICU, intensive care unit; ALT, alanine aminotransferase; AST, aspartate aminotransferase.

The in-ICU and in-hospital mortality rates were 18.6 and 19.6%, respectively. One patient who was discharged alive from the ICU died five days later in a ward. In the nine hospitals selected, 1151 laboratory-confirmed dengue cases occurred with 27 deaths (case fatality rate of 2.3%).

### Factors associated with death

Given that just one patient died after discharge from the ICU, we performed univariate and logistic regression analyses based on in-hospital mortality. Then, the 19 nonsurvivors were compared with the 78 survivors.

Nonsurvivors were older than survivors and more frequently reported hypertension, chronic renal disease, smoking history and a Charlson index value of ≥ 2. Among the warning signs, vomiting, lethargy or restlessness were more common in nonsurvivors. Hemorrhagic bleeding and its variations were not associated with death (Table 3). In-hospital mortality was significantly associated with the APACHE II and SOFA scores.

**Table 3:** Univariate analysis of variables associated with in-hospital mortality among laboratory-confirmed dengue patients admitted to ICUs in Minas Gerais, Brazil.

Variable	Nonsurvivors (n = 19)	Survivors (n = 78)	OR (IC)	*p*
Age (years), mean (SD)	51.7 (18.2)	40.4 (20.4)		0.029
Male sex, n (%)	13 (68.4)	35 (44.9)	2.66 (0.92–7.72)	0.066
Duration of ICU stay (days), median (IR)	2.0 (1.0–6.0)	2.5 (1.0–4.3)		0.532
Duration of hospital stay (days), median (IR)	4.0 (3.0–8.0)	7.0 (4.0–13.3)		0.011
Time from symptoms onset to hospitalization (days), median (IR)	4.0 (3.0–7.0)	4.0 (2.0–6.0)		0.187
Diabetes, n (%)	2 (10.5)	12 (15.4)	0.65 (0.13–3.17)	0.730
Hypertension, n (%)	9 (47.4)	19 (24.4)	2.80 (0.99–7.89)	0.047
Renal chronic disease, n (%)	3 (15.8)	1 (1.3)	14.44 (1.41–147.85)	0.023
Smoking, n (%)	5 (26.3)	4 (5.1)	6.61 (1.58–27.71)	0.013
Charlson index ≥ 2, n (%)	6 (31.6)	9 (11.5)	3.54 (1.08–11.64)	0.030
SOFA, mean (SD)	10.4 (3.3)	4.1 (2.9)		< 0.001
APACHE II, mean (SD)	22.2 (4.9)	9.7 (6.3)		< 0.001
Abdominal pain and/or tenderness, n (%)	13 (68.4)	45 (57.7)	1.59 (0.55–4.62)	0.392
Vomiting, n (%)	13 (68.4)	30 (38.5)	3.47 (1.19–10.10)	0.018
Hepatomegaly, n (%)	3 (15.8)	11 (14.1)	1.14 (0.29–4.58)	1.000
Hypotension and/or syncope, n (%)	11 (57.9)	38 (48.7)	1.45 (0.53–3.99)	0.473
Lethargy or restlessness, n (%)	8 (42.1)	12 (15.4)	4.00 (1.33–12.00)	0.022
Dyspnea or respiratory distress, n (%)	13 (68.4)	29 (37.2)	3.66 (1.26–10.68)	0.014
Cavitary effusion, n (%)	7 (36.8)	21 (26.9)	1.58 (0.55–4.56)	0.392
Clinical edema, n (%)	2 (10.5)	10 (12.8)	0.80 (0.16–4.00)	1.000
Hemorrhagic manifestations (any), n (%)	11 (57.9)	60 (76.9)	0.41 (0.14–1.18)	0.093
Mucosal bleeding, n (%)	5 (26.3)	24 (30.8)	0.80 (0.26–2.48)	0.704
Spontaneous bleeding of the skin, n (%)	7 (36.8)	22 (28.2)	1.49 (0.52–4.26)	0.461
Gastrointestinal bleeding, n (%)	4 (21.1)	18 (23.1)	0.89 (0.26–3.02)	0.850
Hemoglobin (g/dL), mean ± SD	11.4 ± 2.8	11.8 ± 2.3		0.917
Hematocrit (%), mean ± SD	34.2 ± 7.6	34.8 ± 6.7		0.913
Platelet count, mean ± SD	83,338 ± 76,224	98,186 ± 71,973		0.602
Leucocyte count, mean ± SD	12,667 ± 8,313	6,450 ± 5,835		0.024
Cell bands (%), median (IR)	9.3 ± 12.0	4.1 ± 6.0		0.051
Creatinine (mg/dL), median (IR)	1.7 (0.9–2.6)	0.8 (0.6–1.1)		0.007
ALT (U/L), median (IR)	63 (31–157)	47 (30–117)		0.374
AST (U/L), median (IR)	151 (43–474)	67 (31–172)		0.126
Albumin (g/dL), mean ± SD	2.5 ± 0.6	2.9 ± 0.6		0.007
Dengue according to WHO 2009 criteria at ICU admission, n (%)				
Dengue with warning signs	1 (5.3)	29 (37.2)	1	
Severe dengue	19 (94.7)	49 (62.8)	10.7 (1.4–84.0)	0.007
Dengue according to WHO 1997 criteria at ICU admission, n (%)				
Dengue	10 (52.6)	40 (51.3)	1	
Dengue hemorrhagic fever grade I and II	2 (10.6)	25 (32.1)	0.32 (0.06–1.58)	
Dengue hemorrhagic fever grade III and IV	7 (36.8)	13 (16.6)	2.15 (0.68–6.80)	0.062
Cardiorespiratory arrest before ICU, n (%)	2 (10.5)	0 (0.0)	—-	0.037
Vasopressor or inotropic support before ICU, n (%)	7 (36.8)	5 (6.4)	8.52 (2.32–31.25)	< 0.001
Mechanical ventilation before ICU, n (%)	9 (47.4)	6 (7.7)	10.80 (3.17–36.82)	< 0.001
Dialysis during ICU, n (%)	6 (31.6)	6 (7.7)	5.54 (1.55–19.85)	0.005
Vasopressor or inotropic support during ICU, n (%)	16 (84.2)	14 (17.9)	24.38 (6.25–95.18)	< 0.001
Mechanical ventilation during ICU, n (%)	19 (100.0)	10 (12.8)	—-	< 0.001
Red blood cell transfusion, n (%)	5 (26.3)	7 (9.0)	3.62 (1.01–13.06)	0.040
Fresh frozen plasma transfusion, n (%)	5 (26.3)	9 (11.5)	2.74 (0.79–9.41)	0.100
Platelet transfusion, n (%)	4 (21.1)	19 (24.7)	0.83 (0.25–2.80)	0.761
Antibiotic treatment, n (%)	16 (84.2)	28 (35.9)	9.52 (2.55–35.55)	< 0.001
Septic shock, n (%)	9 (47.4)	13 (16.7)	4.50 (1.53–13.25)	0.004
Steroid use, n (%)	5 (26.3)	9 (35.9)	2.74 (0.79–9.41)	0.100

SD, standard deviation; ICU, intensive care unit; IR, interquartile range; SOFA, Sequential Organ Failure Assessment; APACHE, Acute Physiology and Chronic Health Evaluation; ALT, alanine aminotransferase; AST, aspartate aminotransferase; WHO, World Health Organization

The duration of illness preceding hospital admission and the length of time in the ICU were similar between survivors and nonsurvivors. Nonsurvivors spent a shorter length of time in the hospital than that of survivors (Table 3).

Regarding laboratory results, nonsurvivors were found to have lower serum albumin levels, higher serum creatinine levels and higher leucocyte counts compared to survivors. There was a trend of increased cell band percentages among nonsurvivors. Although this continuous variable was not statistically significant, when categorized patients who presented cell band percentages ≥ 10% at ICU admission showed an increased risk of death (OR: 4.47, 95% CI: 1.40–14.30, p = 0.008). Transaminase levels were reported in 85 out of 97 patients (87.6%) upon admission. Three patients demonstrated AST ≥ 1000, and two patients had ALT ≥ 1000. Transaminase levels were similar between survivors and nonsurvivors.

**Fig 1:**
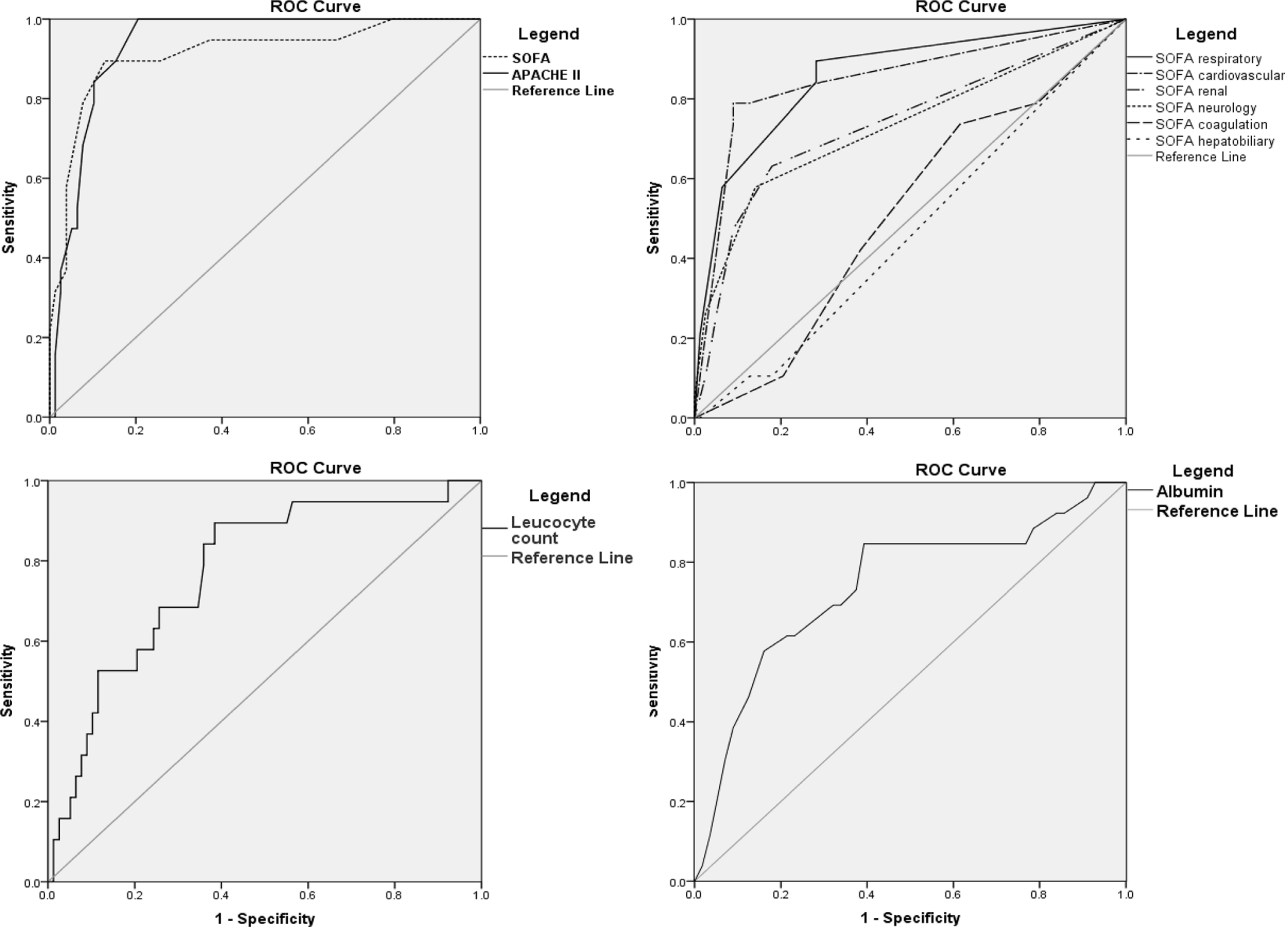
APACHE II score, SOFA score, total leukocyte count and serum albumin concentration AUROCs.

APACHE II and SOFA scores demonstrated excellent areas under the ROC curve (AUROCs) for the prediction of hospital mortality; the APACHE II AUROC was 0.935 (95% CI; 0.887–0.982), and the SOFA AUROC was 0.912 (95% CI; 0.830–0.993) (Fig 1). The respiratory and cardiovascular components of SOFA were the best predictors, whereas the SOFA coagulation and hepatobiliary scores could not be used to discriminate between survivors and nonsurvivors. Hematocrit, platelet counts, AST and ALT ROC curve analysis were not able to discriminate nonsurvivors from survivors. Hematocrit, platelets, AST and ALT each demonstrated AUROC values of 0.499 (95% CI: 0.347–0.651), 0.465 (95% CI: 0.322–0.609), 0.624 (95% CI: 0.463–0.785) and 0.568 (95% CI: 0.406–0.731), respectively. Serum albumin and leucocyte counts were moderately accurate in discriminating survivors from nonsurvivors. The AUROC values were 0.740 (95% CI: 0.576–0.903) and 0.774 (95% CI: 0.658–0.889), respectively. We defined the cutoff for serum albumin and the leucocyte count as a compromise between sensibility and specificity. Patients with serum albumin ≤ 2.8 g/dL demonstrated an 8.5 times increased risk of dying in-hospital (OR: 8.50, 95% CI: 1.72–42.07, p = 0.007), and those with leucocyte counts ≥ 6,000 cells had an 8.53 higher risk of death (OR: 8.53, 95% CI: 2.29–31.78, p < 0.001).

Patients classified as having severe dengue upon ICU admission had a 10.7 times higher risk of dying than did those classified as having dengue with warning signs (Table 3). DHF categories were not associated with death; however, when we analyzed DHF grades III and IV versus DHF grades I and II and DF, there was a trend towards an increased risk of death among the first group (OR: 2.92, 95% CI: 0.96–8.82, p = 0.051).

Among treatments and interventions, inotropic support and mechanical ventilation before and during the ICU stay were associated with death, as was the need for dialysis. Nonsurvivors reported an increased frequency of blood transfusions, antibiotic use and septic shock (Table 3).

Because APACHE II and SOFA demonstrated significant collinearity (Pearson correlation: 0.727, p < 0.001), we performed a multivariate analysis with each score in an independent model. However, none of the other variables that showed statistical significance in the univariate analysis and were included in both models remained in the final model.

Univariate analysis of variables associated with in-ICU mortality is available in S3 Table.

### Dengue WHO classifications

According to the current WHO (2009) classifications, all patients were classified as having dengue with warning signs or severe dengue. Thirty-four (50.7%) out of sixty-seven patients classified as having severe dengue were not classified as DHF, and of those forty-seven dengue patients classified as DHF, fourteen (29.8%) were not classified as having severe dengue (Table 4).

**Table 4:** Comparison between the WHO 1997 dengue classification and WHO 2009 dengue classification for laboratory-confirmed dengue patients admitted to ICUs in Minas Gerais, Brazil.

	DF	DHF I & II	DHF III & IV	Total
	n (%)	n (%)	n (%)	n (%)
Dengue with warning signs	16 (16.5)	14 (14.4)	-	30 (30.9)
Severe dengue	34 (35.1)	13 (13.4)	20 (20.6)	67 (69.1)
Total	50 (51.6)	27 (27.8)	20 (20.6)	97 (100)

DF: dengue fever; DHF I & II: dengue hemorrhagic fever grade I and II; DHF III & IV: dengue hemorrhagic fever grade III and IV.

**Fig 2:**
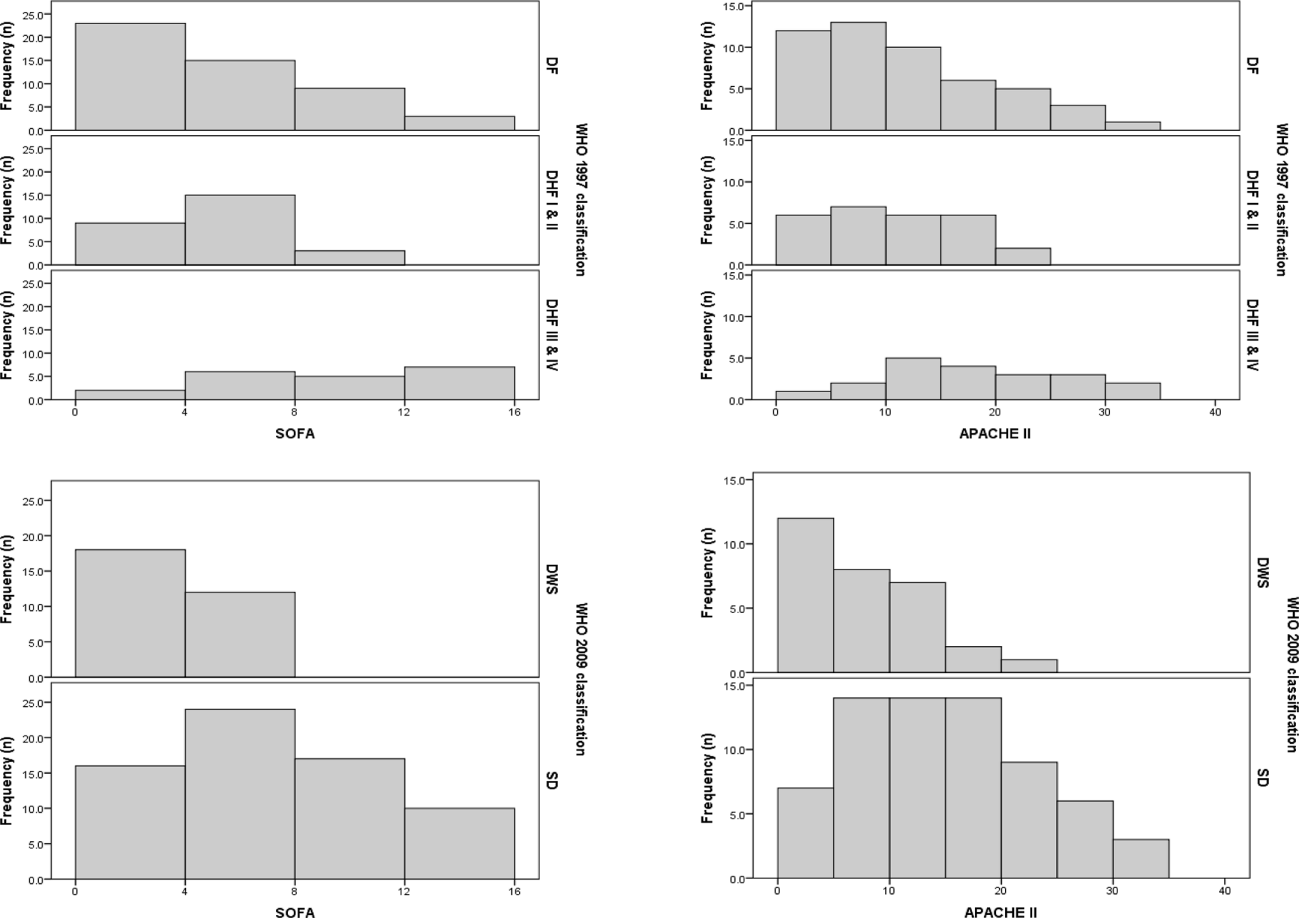
Number of patients in the WHO 1997 and 2009 dengue classifications according to SOFA and APACHE II scores. DF: dengue fever; DHF I & II: dengue hemorrhagic fever grade I and II; DHF III & IV: dengue hemorrhagic fever grade III and IV; DWS: dengue with warning signs; SD: severe dengue.

Both classifications showed a significant overlap in severity grade between their categories (Fig 2). Patients classified as having severe dengue had median and interquartile ranges for SOFA and APACHE II scores of 6.0 (4.0–9.0) and 14.0 (8.0–20.0), respectively, and those classified as having warning signs had values of 3.0 (1.0–4.0) and 6.0 (4.0–10.25), respectively. Patients classified as DF, DHF I and II, and DHF III and IV had median SOFA values of 4.0 (1.0–7.25), 4.0 (3.0–6.0) and 8.5 (5.25–12.75), respectively, and median APACHE II values of 9.5 (4.75–16.0), 10.0 (5.0–15.0) and 16.0 (11.75–24.75), respectively. However, according to the WHO 2009 classifications, all patients with SOFA ≥ 8 were classified as having severe dengue, and 32 of 35 patients (91.4) with APACHE II scores ≥ 15 were classified as having severe dengue (Fig 2).

## Discussion

To the best of our knowledge, our study is one of few to focus on dengue patients admitted to ICUs and the first of these studies to be conducted in Brazil. Furthermore, it is the first to evaluate dengue WHO classifications in the intensive care setting. Unlike other studies that evaluated factors associated with death in general dengue-infected populations [24–26], our evaluation examined a population of dengue patients with a high frequency of organ dysfunction, hemorrhagic manifestations and warning signs.

The in-ICU and in-hospital mortality rates observed in our study were higher than reported by similar studies. Indian studies have reported variable mortalities; Chandralekha [4] and Juneja [6] reported mortalities of 11.1% (8 deaths among 72 patients) and 6.1% (12 deaths among 198 patients), respectively, in similar studies conducted in Indian ICUs. Although the first study did not report severity score data, the latter reported a median APACHE II score of 7.5, which indicate more severe disease in the patients included in our study. The clinical characteristics of our population, such as an increased proportion of patients admitted with shock, respiratory failure, higher severity indices and lower albumin levels, may partially explain the higher mortality. Schmitz study [5], who only included patients who fulfilled the criteria for dengue hemorrhagic fever or dengue shock syndrome, noted a similar mortality rate of 19%. However, other factors could have contributed to our mortality. A recent Brazilian study found a poor adherence to the dengue management protocol among health workers in health units in Brazil. The authors suggested that the deaths analyzed occurred by direct influence of poor quality of the clinical management [27].

Several studies have showed that older dengue patients have an increased risk of dying [28,29]. The increased risk is likely associated with an increased number of comorbid conditions [28] and the progressive deterioration of cardiovascular reserves that occurs during aging [3,27].

Interestingly, leucocyte counts and serum albumin levels were associated with death, whereas hematocrit and platelet count were not. Cell band percentages revealed a trend towards an association with mortality. Low levels of serum albumin have been associated with an increased risk of death for dengue patients and among the general population of critical care patients [30,31]. Hypoalbuminemia is a classical marker of plasma leakage in dengue patients and can be useful as a early sign of severity [30,32]. Regarding leucocyte count, a recent study showed that increased leucocyte and cell band counts were associated with bacterial infection and a higher risk of death in dengue patients [33]. The authors still suggested that leukocytosis and cell band percentages could be used as warning signs in dengue patients [33]. See [34] also found that total leukocyte count is associated with increased risk of bacterial infection. The author even proposed a score, including leucocyte count, to diagnosis concurrent bacterial infection in dengue cases. Our results reinforce the importance of blood cell analysis in critical dengue patients. In addition to evaluating hemoconcentration and decreased platelet counts, the presence of increased leucocyte counts and bands could indicate an increased risk of a non-favorable outcome.

Although several univariate analysis variables were associated with death, none of them remained in the models when APACHE II and SOFA scores were included. It is likely that the high predictive ability of the scores and the low number of events (deaths) limited the performance of those variables in the multivariate models. However, this finding emphasizes the fact that, independent of others possible risk factors, the severity of disease according to APACHE II and organ dysfunction levels upon ICU admission are the main factors associated with death.

In our study, 45 patients received antibiotics, and half were treated for septic shock. It can be difficult to decide between treating bacterial sepsis in dengue shock or dengue with severe organ dysfunction, as the clinical picture for severe sepsis or septic shock can be very similar to that for severe dengue patients [15, 34, 35]. Although delaying antibiotic use may increase the risk of death in possibly coinfected patients, indiscriminate use may increase the risk of multiresistant bacteria [36]. In addition, steroid use could be an additional point of conflict during the treatment of severe dengue cases. Sepsis survival protocols advocate steroids for selected patients with septic shock [36], whereas their use during dengue shock is not recommended due the potential for harm [21]. Dual infection was important because, based on death certificates from the nineteen dengue patients who died during our study, seven (36.8%) presumably died from bacterial infection associated with dengue (S2 Table). Although some studies have addressed these questions regarding septic shock treatment and steroid use in critical dengue patients [34, 35], they remain important issues that should be clarified.

The WHO 2009 dengue classifications are better able to discriminate compared with the previous WHO classifications when comparing nonsurvivors to survivors. The great advantage of the current WHO dengue classifications (WHO 2009) is that they incorporate the definition of any organ dysfunction as a criterion for severe dengue [21], whereas the WHO (1997) dengue classifications considered only cardiovascular dysfunction (hypotension or shock) as the criterion for DHF grade III and IV [20]. Severe dengue case definition, proposed by WHO, 2009, is more similar to the classical criteria of ICU admission which considers any potential or established organ dysfunction (e.g. shock, respiratory and renal failure) for ICU admission. Moreover, a recent study conducted by Pang [37], reported that the WHO 2009 classification of dengue severity was significantly associated with their ICU needs, but not the WHO 1997 classification, which reinforced our findings. However, as shown here, even those patients classified as having severe dengue are part of a continuum that includes patients with low and high risks for death. Despite controversies regarding dengue classification, our data suggest that, among dengue patients who are admitted to ICU, organ failure and the severity index based on APACHE II are more valuable than are the dengue WHO classifications for predicting survival. More studies should address the issue mainly to define if the usual criteria for ICU admission triage could be also applied to dengue cases.

Several studies have shown that comorbid conditions, such as preexisting diabetes mellitus, hypertension and renal chronic disease, are risk factors for death [21, 38–40]. In our study, hypertension, chronic renal disease and Charlson index values of at least 2 were associated with death, as was a history of smoking. Comorbid conditions make dengue management challenging, as fluid management becomes more difficult with comorbidities associated with cardiopulmonary and renal dysfunctions [3, 21].

Our finds reinforce the studies of Juneva [6] and Shmitiz [5], who showed the importance of nonhematologic dysfunction among the factors associated with death in ICU settings. Second to bone marrow suppression and increased peripheral destruction, thrombocytopenia appears to be more of a dengue marker than a real dysfunction predictor of mortality in most critical dengue patients.

With regard to warning signs, our data highlighted the fact that even in critical dengue patients, certain warning signs could be reliable markers of unfavorable outcomes. It is likely that vomiting and lethargy are early markers of associated organ dysfunction. Whereas vomiting could be a sign of splanchnic hypoperfusion, lethargy can be an early sign of neurological dysfunction.

Our study had several limitations. It was conducted only in public hospitals, which may not be representative of the dengue population admitted in ICUs during the time period studied. Furthermore, as a retrospective study, data collected from medical charts may be inadequate due to omitted or inaccurate information. Misclassification due to the complexity of categorizing the participants into specific groups (according to the causes of admission and WHO dengue classifications) is also a limitation. Finally, there is a lack of information regarding treatment administered prior to transfer to the ICU´s hospitals. Most Brazilian dengue patients are treated initially in secondary health units before going to tertiary hospitals where ICU beds are available. In secondary health units the reported information are scarce and not very accurate. Including this information could add a significant bias to the analyses.

We believe that, rather than prioritizing patients with severe dengue for intensive care treatment, earlier admission during the initial phase of organ dysfunction and close monitoring of dengue with warning signs for early recognition of organ failure and hypoperfusion signs could help decrease mortality. Moreover, considering that one of the major functions of the intensive care unit is to provide advanced physiological monitoring to titrate fluids and therapies and avoid fluid overload, intensive monitoring of dengue patients with multiple preexisting comorbidities or limited cardiopulmonary reserves could prevent the progressive deterioration of disease.

In conclusion, the in-ICU and in-hospital mortalities observed in this study were higher than those reported in similar studies. High proportions of ICU admission with higher severity indices and severe organ dysfunction may partially explain the higher mortality reported here. Other factors such as quality of clinical management of the dengue patients and scarcity of ICU beds could have contributed to the higher rate of deceased patients. Prompt ICU admission of severe dengue cases during the early stages of organ dysfunction, continuing education in clinical management of dengue and an increase in the numbers of ICU beds could be important strategies to decrease mortality.

## Supporting Information

S1 Table Number of suspected dengue cases (ages ≥ 15 years old) hospitalized in public health hospitals, admitted to public intensive care units and deceased from 2008–2013 in Minas Gerais, Brazil. (DOCX)

S2 Table Death certificates from 19 dengue patients admitted to intensive care units in Minas Gerais, Brazil. (DOCX)

S3 Table Univariate analysis of variables associated with in-ICU mortality among laboratory-confirmed dengue patients admitted to ICUs in Minas Gerais, Brazil. (DOCX)
