# Supplementary material for: Clinical Profiles and Factors Associated with Death in Adults with Dengue Admitted to Intensive Care Units, Minas Gerais, Brazil
Source: PLoS One. 2015 Jun 19;10(6):e0129046. doi: 10.1371/journal.pone.0129046 (PMC4474920; doi:10.1371/journal.pone.0129046)
Supplement: S1 Table — (DOCX) [file pone.0129046.s001.docx]

Supplementary Table 1 – Number of suspected dengue cases (ages ≥ 15 years old) hospitalized in public health hospitals, admitted to public intensive care units and deceased from 2008-2013 in Minas Gerais, Brazil.

|  | Year | | | | | | |
| --- | --- | --- | --- | --- | --- | --- | --- |
| Variable | 2008 | 2009 | 2010 | 2011 | 2012 | 2013 | Total |
| Patients hospitalized (n) | 1,908 | 2,132 | 5,916 | 1,858 | 1,172 | 6,025 | 19,011 |
| Deaths (n) | 14 | 25 | 51 | 27 | 11 | 55 | 183 |
| Patients admitted to ICU (n) | 35 | 23 | 121 | 32 | 16 | 143 | 370 |
| Deaths among ICU-treated patients (n) | 6 | 2 | 24 | 13 | 5 | 33 | 83 |
| ICU case-fatality rate | 17.1 | 8.7 | 19.8 | 40.6 | 31.3 | 23.1 | 22.4 |
| Dengue suspected patients who died without ICU treatment, n (%) | 8 (57.1) | 23 (92.0) | 27 (52.9) | 14 (51.9) | 6 (54.5) | 22 (40.0) | 100 (54.6) |

Source: Unified Health System (Sistema Único de Saúde, SUS) Hospital Information System (SIH)
