# Supplementary material for: Clinical Profiles and Factors Associated with Death in Adults with Dengue Admitted to Intensive Care Units, Minas Gerais, Brazil
Source: PLoS One. 2015 Jun 19;10(6):e0129046. doi: 10.1371/journal.pone.0129046 (PMC4474920; doi:10.1371/journal.pone.0129046)
Supplement: S2 Table — (DOCX) [file pone.0129046.s002.docx]

Supplementary Table 2 – Death certificates from 19 dengue patients admitted to intensive care units in Minas Gerais, Brazil.

| Cause of death | n (%) |
| --- | --- |
| Shock due to dengue | 4 (21.1) |
| Septic shock from pneumonia due to dengue | 3 (15.8) |
| Cardiogenic shock from myocarditis due to dengue | 2 (10.5) |
| Disseminated intravascular coagulation from shock due to dengue | 1 (5.3) |
| Sepsis from endocarditis due to dengue | 1 (5.3) |
| Sepsis from peritonitis due to dengue | 1 (5.3) |
| Cardiogenic shock from pulmonary embolism due to dengue | 1 (5.3) |
| Hemorrhagic stroke due to dengue | 1 (5.3) |
| Respiratory failure (metabolic acidosis) due to dengue | 1 (5.3) |
| Septic shock | 1 (5.3) |
| Hemorrhagic shock due to dengue in a cirrhotic patient | 1 (5.3) |
| Ischemic stroke due to dengue shock | 1 (5.3) |
| Pneumonia due hemorrhagic stroke due to dengue | 1 (5.3) |
| Total | 19 (100.0) |

Note: The information above refers to the chain of events which led directly to death according to physician´s evaluation. Sepsis implies bacterial infection. Patients whose final cause of death was sepsis, presumed or laboratorial confirmed. For instance “Septic shock due to pneumonia due to dengue” means that the patient was admitted to the ICU with dengue, and, during the course of the disease the patient developed pneumonia and, consequently septic shock.
