# Supplementary material for: Clinical Profiles and Factors Associated with Death in Adults with Dengue Admitted to Intensive Care Units, Minas Gerais, Brazil
Source: PLoS One. 2015 Jun 19;10(6):e0129046. doi: 10.1371/journal.pone.0129046 (PMC4474920; doi:10.1371/journal.pone.0129046)
Supplement: S3 Table — (DOCX) [file pone.0129046.s003.docx]

Supplementary table 3 – Univariate analysis of variables associated with in-ICU mortality among laboratory-confirmed dengue patients admitted to ICUs in Minas Gerais, Brazil.

| **Variable** | Nonsurvivors  (n=19) | Survivors  (n=78) | OR (IC) | *p* |
| --- | --- | --- | --- | --- |
| Age (years), mean (SD) | 50.6 (18.2) | 40.8 (20.4) |  | 0.064 |
| Male sex, n (%) | 12 (66.7) | 36 (45.6) | 2.39 (0.82-7.00) | 0.106 |
| Hypertension, n (%) | 8 (44.4) | 20 (25.3) | 2.36 (0.82-6.81) | 0.106 |
| Renal chronic disease, n (%) | 3 (16.7) | 1 (1.3) | 15.60 (1.52-160.28) | 0.019 |
| Smoking, n (%) | 3 (16.7) | 4 (5.1) | 3.75 (0.76-18.51) | 0.116 |
| Charlson index ≥ 2, n (%) | 6 (33.3) | 9 (11.4) | 3.89 (1.17-12.92) | 0.020 |
| SOFA, mean (SD) | 10.4 (3.3) | 4.1 (2.9) |  | < 0.001 |
| APACHE II, mean (SD) | 22.6 (4.7) | 9.8 (6.3) |  | < 0.001 |
| Abdominal pain and/or tenderness, n (%) | 13 (72.2) | 45 (57.0) | 1.96 (0.64-6.04) | 0.233 |
| Vomiting, n (%) | 13 (72.2) | 30 (38.0) | 4.25 (1.38-13.10) | 0.008 |
| Lethargy or restlessness, n (%) | 7 (38.9) | 13 (16.5) | 3.23 (1.06-9.89) | 0.034 |
| Dyspnea or respiratory distress, n (%) | 12 (66.7) | 30 (38.0) | 3.27 (1.11-9.62) | 0.027 |
| Hemoglobin (g/dL), mean ± SD | 12.1 ± 2.9 | 12.3 ± 2.3 |  | 0.765 |
| Hematocrit (%), mean ± SD | 36.0 ± 7.7 | 36.4 ± 6.6 |  | 0.842 |
| Platelet count, mean ± SD | 85,244 ± 75,553 | 95,312 ± 78,772 |  | 0.623 |
| Leucocyte count, mean ± SD | 12,948 ± 8,461 | 6,464 ± 5,799 |  | 0.006 |
| Creatinine (mg/dL), median (IR) | 1.7 (0.8-2.9) | 0.8 (0.6-1.1) |  | 0.013 |
| Albumin (g/dL), mean ± SD | 2.4 ± 0.5 | 3.0 ± 0.6 |  | 0.002 |
| Dengue according to WHO 2009 criteria at ICU admission, n (%) |  |  |  |  |
| Dengue with warning signs | 1 (5.6) | 29 (36.7) | 1 |  |
| Severe dengue | 17 (94.7) | 50 (63.3) | 9.8 (1.3-77.9) | 0.010 |
| Dengue according to WHO 1997 criteria at ICU admission, n (%) |  |  |  |  |
| Dengue | 9 (50.0) | 41 (51.9) | 1 |  |
| Dengue hemorrhagic fever grade I and II | 2 (11.1) | 25 (31.6) | 0.36 (0.07-1.82) |  |
| Dengue hemorrhagic fever grade III and IV | 7 (38.9) | 13 (16.5) | 2.45 (0.76-7.89) | 0.055 |
| Cardiorespiratory arrest before ICU, n (%) | 2 (11.1) | 0 (0.0) | --- | 0.033 |
| Vasopressor or inotropic support before ICU, n (%) | 7 (38.9) | 5 (6.3) | 8.42 (2.54-34.90) | < 0.001 |
| Mechanical ventilation before ICU, n (%) | 8 (44.4) | 7 (8.9) | 14.19 (2.45-27.62) | < 0.001 |
| Dialysis during ICU, n (%) | 6 (33.3) | 6 (7.6) | 6.08 (1.68-22.00) | 0.003 |
| Vasopressor or inotropic support during ICU, n (%) | 15 (83.3) | 15 (19.0) | 21.33 (5.47-83.20) | < 0.001 |
| Mechanical ventilation during ICU, n (%) | 18 (100.0) | 11 (13.9) | --- | < 0.001 |
| Red blood cell transfusion, n (%) | 5 (27.8) | 7 (8.9) | 3.96 (1.09-14.38) | 0.028 |
| Fresh frozen plasma transfusion, n (%) | 5 (27.8) | 9 (11.4) | 2.99 (0.86-10.37) | 0.074 |
| Platelet transfusion, n (%) | 4 (22.2) | 19 (24.1) | 0.90 (0.27-3.07) | 0.869 |
| Antibiotic treatment, n (%) | 15 (83.3) | 29 (36.7) | 8.62 (2.30-32.31) | < 0.001 |
| Septic shock, n (%) | 9 (50.0) | 13 (16.5) | 5.08 (1.69-15.23) | 0.002 |
| Steroid use, n (%) | 5 (27.8) | 9 (11.4) | 2.99 (0.86-10.37) | 0.074 |

SD, standard deviation; ICU, intensive care unit; IR, interquartile range; SOFA, Sequential Organ Failure Assessment; APACHE, Acute Physiology and Chronic Health Evaluation; WHO, World Health Organization
